# Sleep Phenotypes, Genetic Susceptibility, and Risk of Obesity in Patients With Type 2 Diabetes: A National Prospective Cohort Study: A Commentary

**DOI:** 10.1111/1753-0407.70146

**Published:** 2025-08-15

**Authors:** Nimra Khan, Rida‐e‐Zehra, Nandni Dileep

**Affiliations:** ^1^ Liaquat University of Medical and Health Sciences Jamshoro Pakistan

**Keywords:** genetic susceptibility, obesity, sleep phenotypes, type 2 diabetes

## Abstract

This commentary discusses the study “Sleep Phenotypes, Genetic Susceptibility, and Risk of Obesity in Patients with Type 2 Diabetes: A National Prospective Cohort Study” by Xi et al. The study provides a perceptive investigation of how metabolic characteristics and genetic variables interact to affect body mass index (BMI) in people with T2D. It has some limitations, including reliance on self‐reported sleep data and lack of in‐depth sleep quality assessment. We highlight the importance of objective sleep data, detailed assessments of sleep quality, and advanced polygenic risk models in future research to better understand these relationships and inform precision diabetes care.

1

**Summary**

- The study looks into how sleep patterns, genetic risk, and obesity risk are linked in people with T2D.
- Limitations include sleep data that people report themselves and a lack of a full sleep quality assessment.
- Future study should use objective sleep data and more refined polygenic risk models.

To the Editor,

1

The study “Sleep Phenotypes, Genetic Susceptibility, and Risk of Obesity in Patients with Type 2 Diabetes (T2D): A National Prospective Cohort Study” by Xi et al. [1] caught our attention. The study provides a perceptive investigation of how metabolic characteristics and genetic variables interact to affect body mass index (BMI) in people with T2D.

Despite the fact that the study offers insightful information, a few limitations should be noted. First of all, measurement inaccuracies may be introduced because it depends on self‐reported sleep data. This problem was resolved in a cited study that produced more reliable data by objectively measuring habitual sleep duration using actigraphy [2].

Moreover, sleep quality is not fully taken into consideration in the study. By involving a thorough assessment of sleep quality, the article “Sleep Quality and Associated Factors in Adults with Type 2 Diabetes: A Retrospective Cohort Study” overcomes the parent article's flaw. Particularly, it uses proven instruments, the Pittsburgh Sleep Quality Index (PSQI) and the Epworth Sleepiness Scale (ESS), to assess both immoderate daytime sleepiness and the quality of sleep at night. By using this approach, the study addresses the confounding element of sleep quality that was overlooked in the previous research and offers a more thorough knowledge of sleep problems in people with type 2 diabetes [3].

Obesity and sleep relationship have been linked in studies; however, the root causes are still undetermined. One crucial drawback is that there aren't various long‐term sleep restriction research studies; the majority last less than 3 weeks, so the long‐term effects are unknown. There is also a lack of research on napping, obesity, and weight gain, especially in those with type 2 diabetes (T2D). It pulls out attention to a larger problem with the parent article: it overlooks how sleep issues affect metabolic health. This is addressed in the mentioned review, which stresses the need for diagnosing and treating sleep disturbances in people with type 2 diabetes because they are more frequent in this population, may worsen outcomes, and accelerate the progression of the disease. Taking care of sleep issues could enhance T2D care and provide information that the parent article missed [4].

The parent study may have skipped intricate genetic factors altering BMI because it only used a restricted number of genetic variants for creating the BMI polygenic risk score (PRS). Key variables such as socioeconomic status, physical activity, and food were not taken to notice. On the other hand, present‐day PRS methods employ advanced statistical frameworks to precisely capture genetic complexity, offering an improved understanding of genetic risk related to BMI [5].

Despite its limitations, the parent article offers valuable details about behavioral and genetic factors affecting the risk of obesity in people with type 2 diabetes. To better understand these relationships and inform precision diabetes care, future research implementing objective sleep data, detailed assessments of sleep quality and disorders, and advanced polygenic risk models is crucial.

## Author Contributions

**Nimra Khan:** conceptualization, methodology, writing original draft, writing review, and editing. **Rida‐e‐Zehra:** data analysis, resources, writing original draft, and editing. **Nandni Dileep:** investigation, writing original draft, writing review, and editing.

## Disclosure

The authors have nothing to report.

## Ethics Statement

The authors have nothing to report.

## Conflicts of Interest

The authors declare no conflicts of interest.

## Data Availability

The authors have nothing to report.

## References

[1] L. Xi , L. Li , S. Fu , et al., “Sleep Phenotypes, Genetic Susceptibility, and Risk of Obesity in Patients With Type 2 Diabetes: A National Prospective Cohort Study,” Journal of Diabetes 17, no. 5 (2025): e70095, 10.1111/1753-0407.70095. 40394863 PMC12092374

[2] S. Javaheri , A. Storfer‐Isser , C. L. Rosen , and S. Redline , “Association of Short and Long Sleep Durations With Insulin Sensitivity in Adolescents,” Journal of Pediatrics 158, no. 4 (2011): 617–623, 10.1016/j.jpeds.2010.09.080. 21146189 PMC3076647

[3] C. P. Kuo , S. H. Lu , C. N. Huang , W. C. Liao , and M. C. Lee , “Sleep Quality and Associated Factors in Adults With Type 2 Diabetes: A Retrospective Cohort Study,” International Journal of Environmental Research and Public Health 18, no. 6 (2021): 3025, 10.3390/ijerph18063025. 33804208 PMC7999598

[4] S. B. J. Schipper , M. M. Van Veen , P. J. M. Elders , et al., “Sleep Disorders in People With Type 2 Diabetes and Associated Health Outcomes: A Review of the Literature,” Diabetologia 64, no. 11 (2021): 2367–2377, 10.1007/s00125-021-05541-0. 34401953 PMC8494668

[5] D. Jayasinghe , S. Eshetie , K. Beckmann , B. Benyamin , and S. H. Lee , “Advancements and Limitations in Polygenic Risk Score Methods for Genomic Prediction: A Scoping Review,” Human Genetics 143, no. 12 (2024): 1401–1431, 10.1007/s00439-024-02716-8. 39542907

